# Altered Secretion, Constitution, and Functional Properties of the Gastrointestinal Mucus in a Rat Model of Sporadic Alzheimer’s Disease

**DOI:** 10.1021/acschemneuro.3c00223

**Published:** 2023-07-21

**Authors:** Jan Homolak, Joke De Busscher, Miguel Zambrano-Lucio, Mihovil Joja, Davor Virag, Ana Babic Perhoc, Ana Knezovic, Jelena Osmanovic Barilar, Melita Salkovic-Petrisic

**Affiliations:** † Department of Pharmacology, University of Zagreb School of Medicine, 10 000 Zagreb, Croatia; ‡ Croatian Institute for Brain Research, University of Zagreb School of Medicine, 10 000 Zagreb, Croatia; § Catholic University of Leuven, 3000 Leuven, Belgium; ∥ School of Medicine, Autonomous University of Nuevo Leon, Monterrey, Nuevo Leon 66455, Mexico; ⊥ Department of Infection and Immunity, Luxembourg Institute of Health, L-4354 Esch-sur-Alzette, Luxembourg; # Faculty of Science, Technology and Medicine, University of Luxembourg, L-4365 Esch-sur-Alzette, Luxembourg

**Keywords:** Alzheimer’s disease, streptozotocin, brain−gut axis, mucus, neurodegeneration

## Abstract

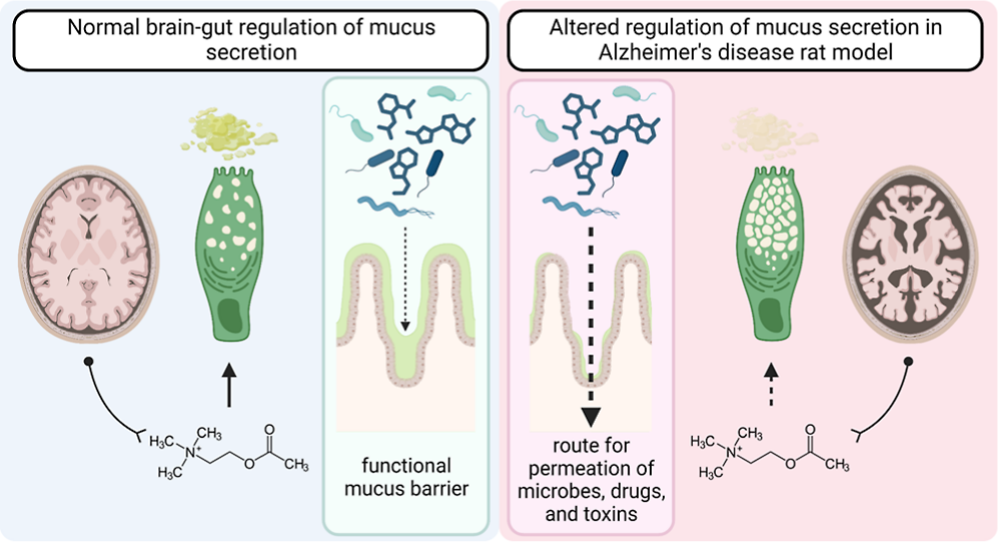

The gastrointestinal (GI) system is affected in Alzheimer’s disease (AD); however, it is currently unknown whether GI alterations arise as a consequence of central nervous system (CNS) pathology or play a causal role in the pathogenesis. GI mucus is a possible mediator of GI dyshomeostasis in neurological disorders as the CNS controls mucus production and secretion via the efferent arm of the brain–gut axis. The aim was to use a brain-first model of sporadic AD induced by intracerebroventricular streptozotocin (STZ-icv; 3 mg/kg) to dissect the efferent (i.e., brain-to-gut) effects of isolated central neuropathology on the GI mucus. Morphometric analysis of goblet cell mucigen granules revealed altered GI mucus secretion in the AD model, possibly mediated by the insensitivity of AD goblet cells to neurally evoked mucosal secretion confirmed by ex vivo cholinergic stimulation of isolated duodenal rings. The dysfunctional efferent control of the GI mucus secretion results in altered biochemical composition of the mucus associated with reduced mucin glycoprotein content, aggregation, and binding capacity in vitro. Finally, functional consequences of the reduced barrier-forming capacity of the mucin-deficient AD mucus are demonstrated using the in vitro two-compartment caffeine diffusion interference model. Isolated central AD-like neuropathology results in the loss of efferent control of GI homeostasis via the brain–gut axis and is characterized by the insensitivity to neurally evoked mucosal secretion, altered mucus constitution with reduced mucin content, and reduced barrier-forming capacity, potentially increasing the susceptibility of the STZ-icv rat model of AD to GI and systemic inflammation induced by intraluminal toxins, microorganisms, and drugs.

## Introduction

Accumulating evidence suggests that the gastrointestinal (GI) tract plays a role in Alzheimer’s disease (AD): (i) the GI symptoms are more prevalent in patients diagnosed with AD than in control populations;^1^ (ii) patients with inflammatory bowel disease are at an increased risk of developing AD;^2,3^ (iii) there seems to be an overlap in genetic traits mediating susceptibility to AD and some GI disorders (e.g., gastroesophageal reflux disease, gastritis-duodenitis, peptic ulcer disease, and diverticulosis);^4^ (iv) intestinal microbiota of AD patients differs from that obtained from healthy controls;^5−7^ (v) animal studies support the existence of pathophysiological mechanisms by which GI perturbations may lead to neurodegeneration (e.g., the development of cerebral amyloidosis and cognitive impairment following retrograde transport of intra-GI administration of amyloid-β (Aβ) oligomers;^8^ gut dyshomeostasis-induced inflammation and metabolic dysfunction-driven neurodegeneration^9−12^).

It is currently unknown whether GI alterations primarily arise as a consequence of central nervous system (CNS) pathology or play a causal role in the pathogenesis of the disease. Either way, it is reasonable to assume that GI dyshomeostasis acts as an important pathophysiological factor, as loss of physiological functions of the gut (absorption of nutrients, maintenance of the immunological and physical barrier to foreign substances and microorganisms) inevitably leads to inflammation and metabolic dysfunction with the potential to initiate and/or promote neurodegeneration.^9−13^ The GI mucus system may play an important role in the pathophysiology of the gut–brain axis dysfunction in AD as mucus homeostasis is regulated by the nervous system (e.g., acetylcholine (ACh)-mediated neurally evoked mucosal secretion and GI motility and peristalsis-mediated regulation of mucus renewal).^14^ GI mucus is integral to gut health because it acts as the first line of defense against luminal contents (i.e., residing and exogenous microorganisms, orally ingested toxins, digestive enzymes, acid, etc.). Consequently, exogenous (e.g., microorganisms, drugs, and toxins) and endogenous (loss/dysfunction of neural control) factors that alter its structural and functional properties bear the potential to place a substantial burden on organismic homeostasis and promote inflammation and neurodegeneration.

Pathophysiological alterations of the GI tract are present in both non-transgenic^15−17^ and trangenic^18−20^ animal models of AD; however, not much is known about the involvement of GI mucus. Honarpisheh et al. observed reduced GI mucus fucosylation (reflecting mucus maturation) in the Tg2576 model,^18^ and Ou et al. reported a reduction in colonic goblet cell number in the APP/PS1 transgenic mice.^21^ In Tg2576, reduced mucus maturation was associated with a breached epithelial barrier, altered GI absorption, accumulation of GI Aβ, and increased inflammation.^18^ Furthermore, GI alterations and peripheral inflammation were present before the accumulation of cerebral Aβ, suggesting cauda-rostral propagation.^18^

Considering the importance of mucus for the maintenance of GI homeostasis and its possible role in the pathogenesis of neurodegeneration,^14^ we explored the GI mucus system in the rat model of sporadic AD induced by intracerebroventricular streptozotocin (STZ-icv). The focus on the GI mucus in the STZ-icv model was based on the following: (i) the STZ-icv is a “brain-first” model (i.e., targeted application of the toxin ensures that pathophysiological alterations are spatiotemporally defined and localized to the CNS following model induction) that successfully mimics many aspects of AD-related neuropathology (i.e., cognitive deficits,^22^ neuroinflammation,^23^ Aβ accumulation,^24^ tau hyperphosphorylation,^25^ glucose hypometabolism,^26^ insulin-resistant brain state,^27^ oxidative stress,^28^ and mitochondrial dysfunction^27^). Consequently, unlike transgenic animals (except for tissue-specific inducible models), the STZ-icv model is appropriate for dissecting the efferent (i.e., brain-to-gut) effects of central neuropathology mediated by the brain–gut axis; (ii) the STZ-icv GI barrier is characterized by structural and functional alterations associated with dysfunctional duodenal epithelial cell turnover and apoptosis.^16,17^ Altered function of the GI mucus leads to increased exposure of the epithelium to intraluminal noxious stimuli, which may result in suppression of apoptosis (a GI integrity-compatible cell death pathway) and activate inflammation-associated cell death pathways (e.g., pyroptosis or necroptosis).^16,29^ Furthermore, dysfunctional regulation of epithelial cell death and shedding may result in the loss of mucus-producing goblet cells; (iii) redox homeostasis, an important etiopathogenetic factor in AD,^16,30^ is impaired in the brain,^28^ plasma,^15,31^ and the gut^15,17^ in the STZ-icv rat model of sporadic AD. Redox balance is important for the maintenance of normal functioning of the GI barrier and the mucus layer;^32−34^ (iv) STZ-icv GI tract seems to be unresponsive to central pharmacological modulation of glucagon-like peptide-1 (GLP-1) and glucose-dependent insulinotropic polypeptide (GIP) receptors, suggesting that the communication between the brain and the gut may be impaired.^15,16^ The intact gut–brain axis seems to be important for the maintenance of the GI mucus layer, as mucus secretion is under neural control.^14^

Based on all of the above, we hypothesized that mucus secretion and homeostasis may be altered in the GI tract of the STZ-icv rat model of AD.

## Results

The STZ-icv duodenal epithelium is characterized by an increased number of goblet cells with possibly altered mucus secretion.

**Figure 1:**
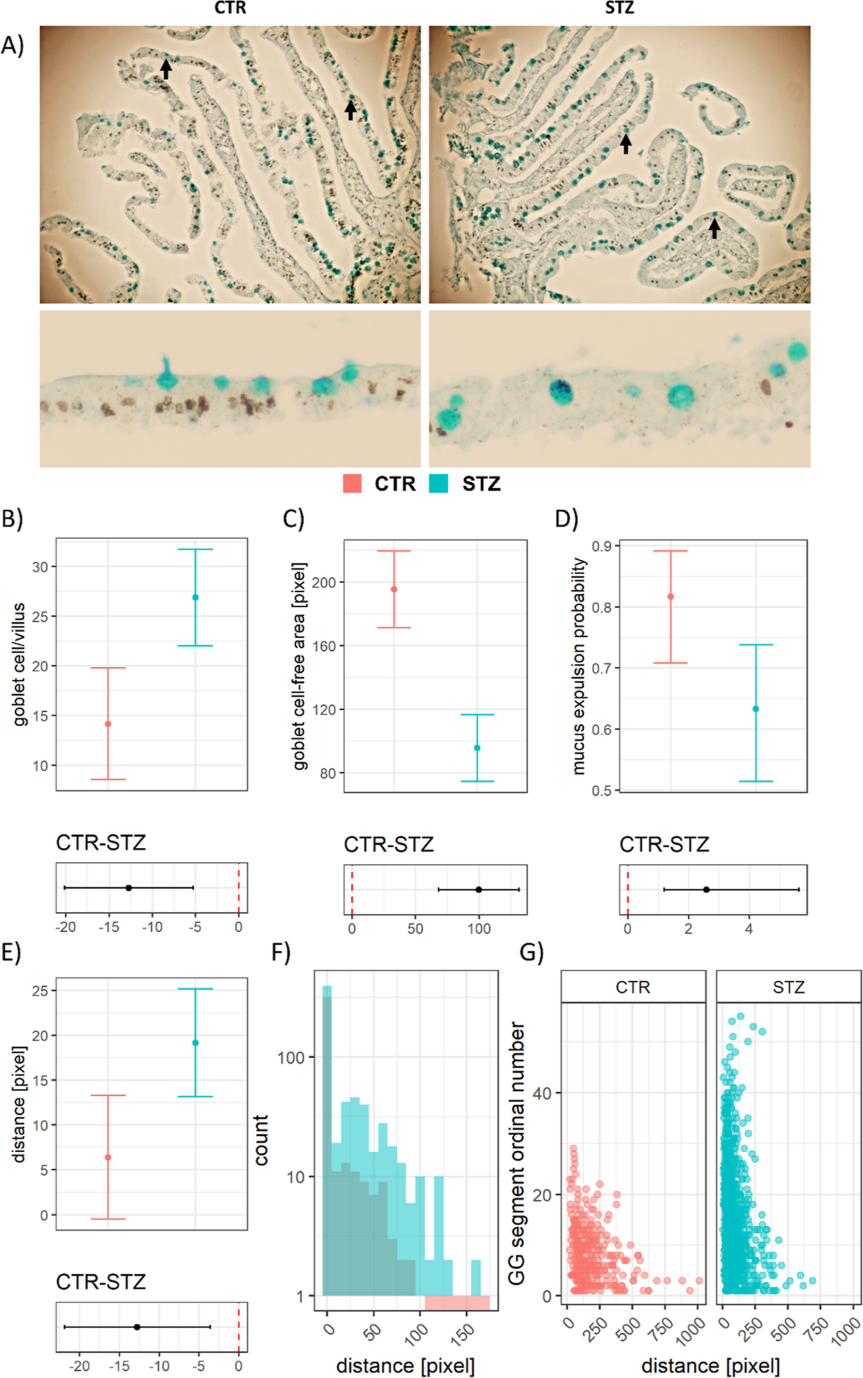
Quantitative analysis of alcianophilic vesicles in the duodenal mucosa of the rat model of AD induced by intracerebroventricular streptozotocin (STZ-icv). (A) Representative photomicrographs of the duodenal mucosa from the STZ-icv and control animals demonstrating an increased number of mucinous vesicles (black arrows) in the STZ-icv mucosa (upper) with fewer vesicles undergoing mucus expulsion (lower). (B) Model-derived estimates from the linear mixed model reflecting the number of goblet cells/villus in the STZ-icv and the controls (upper) and the contrast illustrating the effect size (lower). Mean estimates are accompanied by 95% confidence intervals. (C) Model-derived estimates from the linear mixed model reflecting the epithelial surface between adjacent goblet cells in the STZ-icv and the controls (upper) and the contrast illustrating the effect size (lower). Mean estimates are accompanied by 95% confidence intervals. (D) Model-derived estimates from the mixed effects logistic regression model reflecting the probability of mucus expulsion in the STZ-icv and the controls (upper) and the contrast illustrating the effect size (lower). Mean estimates are accompanied by 95% confidence intervals. (E) Model-derived estimates from the linear mixed model reflecting the distance between the mucus-containing vesicles and the epithelial surface in the STZ-icv and the controls (upper) and the contrast illustrating the effect size (lower). Mean estimates are accompanied by 95% confidence intervals. (F) Histogram of the number of mucinous granules with respect to distance from the epithelial surface in the control and STZ-icv-treated rats. (G) Association between the ordinal number of the mucosal segment (starting from the tip of the villus) and the distance of mucinous granules from the epithelial surface in the controls and the STZ-icv rat model of AD.

Analysis of the Alcian blue (AB)-stained duodenal tissue sections from the in vivo experiment demonstrated an increased number of goblet cells in the STZ-icv rat model of AD (Figure 1A) with the difference being most pronounced in the upper portion of the villi. Interestingly, upon closer inspection, goblet cell mucin-containing secretory vesicles were also further away from the epithelial surface, and there were fewer vesicles undergoing expulsion in the STZ-icv rats, suggesting that apparent goblet cell hyperplasia may be due to dysfunctional secretion of vesicles and not necessarily a reflection of increased generation and/or suppressed cell apoptosis/extrusion. A linear mixed effects model of goblet cell count indicated that there were ∼90% more (∼27 vs 14 cells/villus) goblet cells in the STZ-icv duodenum after accounting for repeated sampling (Figure 1B). An alternative measure (to account for possible differences in villus length in the STZ-icv^16^) revealed that the goblet cell-free area (modeled as pixel distance between two AB-positive vesicles) was ∼2-fold greater in the controls (195 pixels in the CTR vs 95 pixels in the STZ-icv) (Figure 1C). To check whether the dysfunctional mechanism of secretory vesicle expulsion provides a possible explanation for the observed phenomenon, a mixed effects logistic regression model was used to calculate the probability of mucus secretion. The model revealed that there was a greater probability of mucus expulsion in the goblet cells of the controls in comparison with the STZ-icv (82 vs 63%; OR 2.59 [1.19–5.63]; *p* = 0.02) (Figure 1D). Considering that mucigen granules travel toward the luminal surface of the cell to undergo exocytosis, the distance between the luminal border of the secretory vesicle, and the epithelial surface was analyzed as an additional indicator of dysfunctional secretion/expulsion, showing that vesicles from the STZ-icv goblet cells were on average ∼3-fold further away from the surface (Figure 1E). Histograms illustrating the number of AB-positive granules vs the distance from the epithelial surface show that regardless of the fact that there were ∼25% more granules touching the epithelial surface with the luminal border in the STZ-icv rats, there was also a greater number of distant secretory granules with a reduced probability of expulsion (Figure 1F). Finally, the number of secretory granules was shown in terms of goblet cell-free epithelial surface against the position along the villus axis (starting from the tip of the villus), supporting the working hypothesis that mucus secretion was altered in the STZ-icv (Figure 1G).

Goblet cells from the STZ-icv duodenum demonstrate reduced responsiveness to cholinergic stimulation ex vivo.

**Figure 2:**
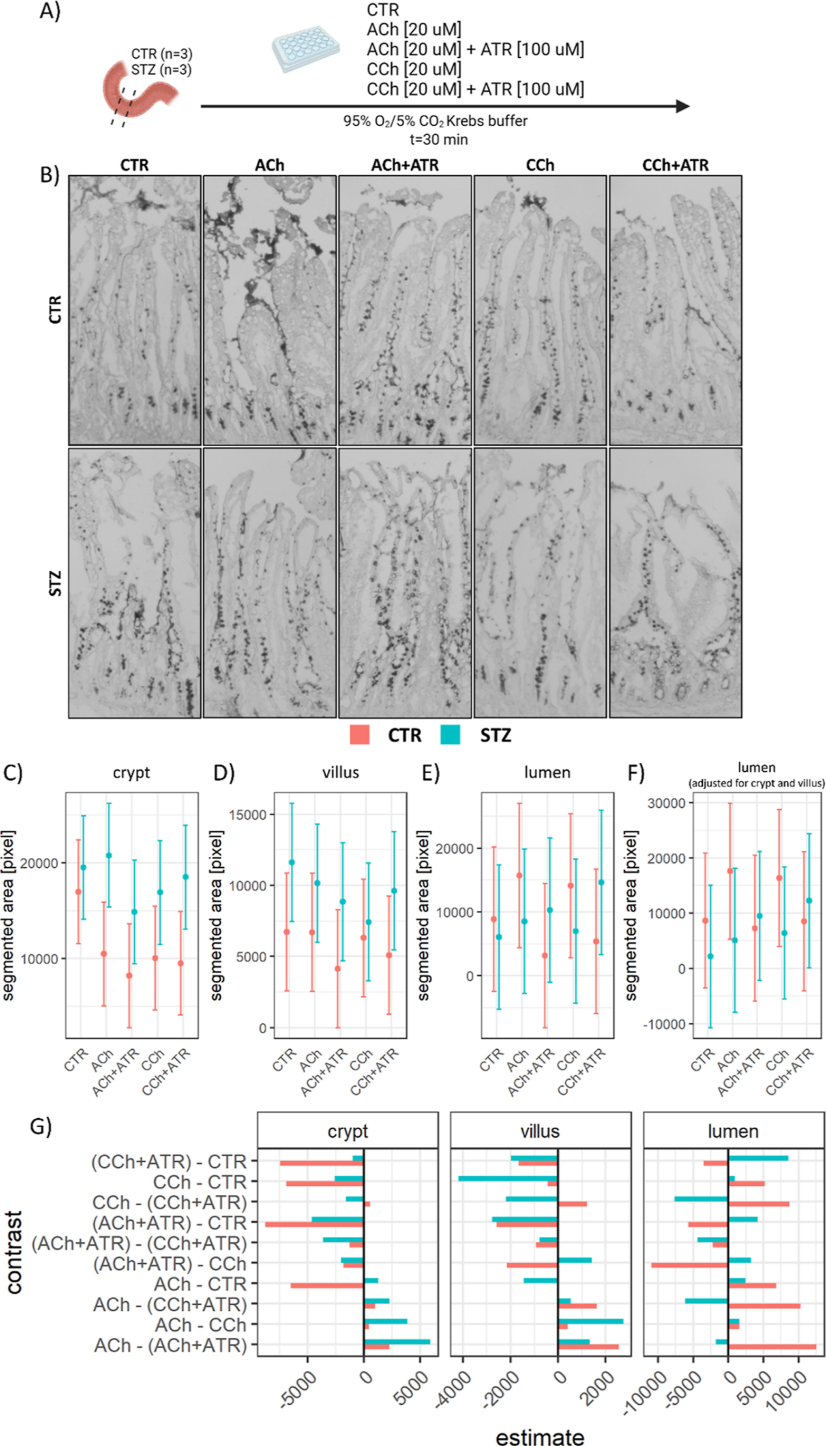
Results from the ex vivo experiment designed to test the sensitivity of duodenal goblet cells of the control animals and the animal model of AD [induced by intracerebroventricular streptozotocin (STZ-icv)] to the cholinergic stimulation-induced secretion of GI mucus. (A) Experimental design: Duodenal tissue from the control (*n* = 3) and the STZ-icv rat model of AD (*n* = 3) was dissected and incubated with the control Krebs buffer (CTR); Krebs buffer + 20 μM ACh; Krebs buffer + 20 μM ACh + 100 μM atropine (ACh + ATR); Krebs buffer + 20 μM carbachol (CCh); and Krebs buffer + 20 μM carbachol + 100 μM atropine (CCh + ATR). (B) Representative photomicrographs showing mucosal and intraluminal alcianophilic signals (mucus) 30 min after incubation. (C) Model-derived estimates from the linear mixed model reflecting the crypt mucus (estimated based on segmented area) in the STZ-icv and the controls (upper) and the contrast illustrating the effect size (lower). Mean estimates are accompanied by 95% confidence intervals. (D) Model-derived estimates from the linear mixed model reflecting the villus mucus (estimated based on segmented area) in the STZ-icv and the controls (upper) and the contrast illustrating the effect size (lower). Mean estimates are accompanied by 95% confidence intervals. (E) Model-derived estimates from the linear mixed model reflecting luminal mucus (estimated based on segmented area) in the STZ-icv and the controls (upper) and the contrast illustrating the effect size (lower). Mean estimates are accompanied by 95% confidence intervals. (F) Model-derived estimates from the linear mixed model reflecting luminal mucus adjusted for the crypt and villus mucus content (estimated based on segmented area) in the STZ-icv and the controls (upper) and the contrast illustrating the effect size (lower). Mean estimates are accompanied by 95% confidence intervals. (G) Contrasts of mean estimates from the models reported in (C–E).

To test the hypothesis that STZ-icv duodenal goblet cells are characterized by dysfunctional secretion of mucigen granules, the tissue was subjected to cholinergic stimulation ex vivo (Figure 2A). The incubation of duodenal tissue with two different cholinergic agonists (ACh and CCh) stimulated mucus secretion in the controls, but not in the STZ-icv rat model of AD (Figure 2B). Co-incubation with a competitive, reversible antagonist of the muscarinic ACh receptors (ATR) prevented the cholinergic stimulation-induced mucus secretion observed in the control animals (Figure 2B). The ATR-treated tissue also demonstrated a subtle reduction in mucus secretion from baseline (i.e., in comparison with control samples) accompanied by an increased number of inert mucigen granules in the upper segment of the villus in both groups (Figure 2B); however, the effect was challenging to quantify with certainty due to substantial anatomical variation among villi (Figure 2C–E). Quantitative analysis demonstrated a 38 and 41% reduction in the AB-positive crypt area upon stimulation with ACh and CCh, respectively, in the controls, while the same treatment was associated with a 7% increase and a 13% reduction in the STZ-icv (Figure 2C). In the villus region, ACh and CCh had no effect on the AB-positive area, while ATR was associated with a 31% reduction on average in the controls. In contrast, all treatments were associated with a reduction of segmented AB-positive area (ACh: −12%; ACh + ATR: −24%; CCh: −36%; and CCh + ATR: −17%) in the STZ-icv-treated rats (Figure 2D). Quantification of estimated luminal AB-positive content revealed a pattern of increased secretion upon cholinergic stimulation (ACh: +77%; CCh: +59%) successfully prevented/reversed by co-incubation with ATR (ACh + ATR: −64%; CCh + ATR: −39%) in the controls (Figure 2E). In contrast, in the rat model of AD, the impact of cholinergic stimulation was significantly less pronounced. Specifically, the administration of ACh resulted in a 41% increase, while CCh led to a 15% increase in the estimated luminal mucus concentration (Figure 2E). Interestingly, when co-incubated with ATR, which blocks cholinergic receptors, there was an increase rather than a decrease in the estimated luminal mucus concentration (ACh + ATR: +70%; CCh + ATR: +142%) (Figure 2E). Furthermore, we confirmed the same pattern of cholinergic stimulation-mediated mucus secretion sensitive to atropine in the control group but the absence of this effect in the STZ-icv AD model. We employed a model in which the luminal signal was adjusted for the content of mucin in the crypts and villi (Figure 2F) to validate these findings. The contrasts between mean estimates have been reported for easier comparisons (Figure 2G).

Impaired goblet cell function is associated with altered content and functional properties of mucus.

**Figure 3:**
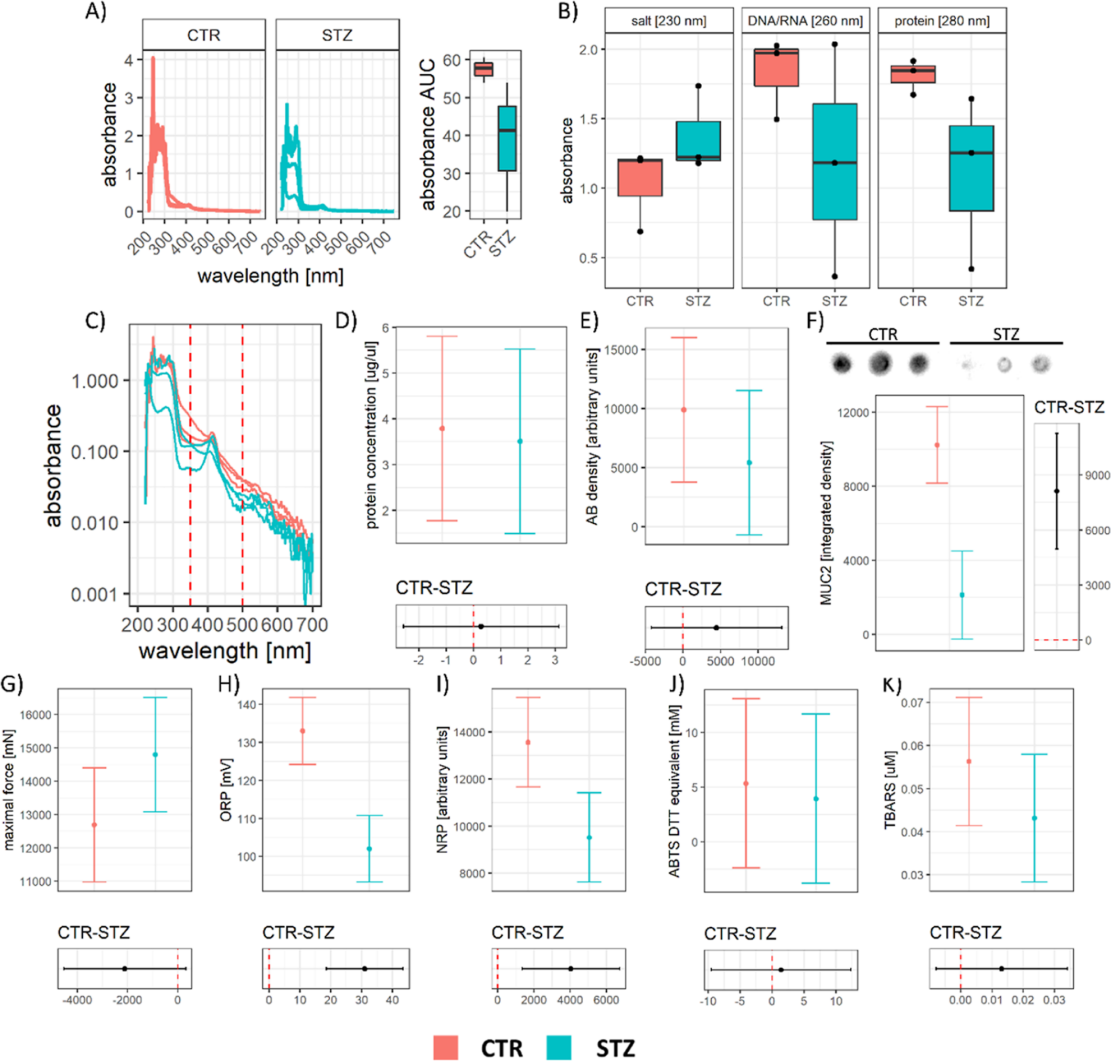
Biochemical analysis of the GI mucus obtained from the rat model of sporadic AD induced by intracerebroventricular streptozotocin (STZ-icv) and the controls. (A) UV–vis spectra of mucus from the control and the STZ-icv animals (left) and the area under the curve reflecting dilution (right). (B) Absorbance of samples at 230, 260, and 280 nm, reflecting the concentration of salts, nucleic acids, and proteins. (C) Relationship between sample absorbance (log-transformed *y*-axis) and wavelength depicted for the demonstration of the relationship between the 400 nm peak and the UV region. (D) Model-derived estimates from the linear model reflecting the concentration of protein in the mucus of the STZ-icv and the controls (upper) and the contrast illustrating the effect size (lower). Mean estimates are accompanied by 95% confidence intervals. (E) Model-derived estimates from the linear model reflecting the signal intensity of AB; reflecting glycoprotein content) in the mucus of the STZ-icv and the controls (upper) and the contrast illustrating the effect size (lower). Mean estimates are accompanied by 95% confidence intervals. (F) Mucin 2 (MUC2) dot blot, model-derived estimates from the linear model reflecting the concentration of MUC2 in the mucus of the STZ-icv and the controls (left) and the contrast illustrating the effect size (right). Mean estimates are accompanied by 95% confidence intervals. (G) Model-derived estimates from the linear model reflecting peak mastPASTA-obtained force (inversely correlated with lubrication capacity) in the mucus of the STZ-icv and the controls (upper) and the contrast illustrating the effect size (lower). Mean estimates are accompanied by 95% confidence intervals. (H) Model-derived estimates from the linear model reflecting the oxidation–reduction potential (ORP) in the mucus of the STZ-icv and the controls (upper) and the contrast illustrating the effect size (lower). Mean estimates are accompanied by 95% confidence intervals. (I) Model-derived estimates from the linear model reflecting the NRP-integrated density in the mucus of the STZ-icv and the controls (upper) and the contrast illustrating the effect size (lower). Mean estimates are accompanied by 95% confidence intervals. (J) Model-derived estimates from the linear model reflecting the 2,2′-azino-bis(3-ethylbenzothiazoline-6-sulfonic acid (ABTS)-derived reductive capacity (in equivalents of dithiothreitol [mM]) in the mucus of the STZ-icv and the controls (upper) and the contrast illustrating the effect size (lower). Mean estimates are accompanied by 95% confidence intervals. (J) Model-derived estimates from the linear model reflecting lipid peroxidation (estimated using the thiobarbituric acid reactive substances (TBARS) assay) in the mucus of the STZ-icv and the controls (upper) and the contrast illustrating the effect size (lower). Mean estimates are accompanied by 95% confidence intervals.

The STZ-icv mucus was analyzed to examine whether the observed impairment of goblet cell function was associated with functional changes that may play a role in the GI redox dyshomeostasis reported in the STZ-icv model of AD.^15,16^ The UV–vis spectra of mucus revealed quantitative differences, with STZ-icv mucus demonstrating reduced average absorbance across the spectrum (Figure 3A). The analysis of individual wavelengths in the UV region suggested that STZ-icv mucus had fewer nucleic acids and proteins (Figure 3B). Furthermore, the spectral analysis indicated a possible difference in other molecules, as evident from ratiometric differences in specific spectral regions [e.g., the 400 nm peak in relation to the UV region (Figure 3C)]. Interestingly, the protein content determined by the Bradford method showed no pronounced differences between the mucus of the controls, and the STZ-icv model suggests that some other molecule(s) may be responsible for the difference in the 280 nm absorbance (Figure 3D). On the other hand, AB and mucin 2 (MUC2) dot blots revealed a ∼50% reduction of the alcianophilic content and ∼80% reduction of MUC2 in the STZ-icv mucus (Figure 3E,F). Reduced AB and MUC2 content were in concordance with perturbed biotribometric properties of the STZ-icv mucus, as it demonstrated reduced lubricating capacity when compared with the mucus obtained from the controls (Figure 3G). Finally, redox-related biomarkers were measured to examine whether the observed changes were associated with altered local redox homeostasis. The total antioxidant capacity measured by oxidation-reduction potential (ORP) and nitrocellulose redox permanganometry (NRP) provided contradictory information, with ORP suggesting an increased and NRP suggesting a decreased antioxidant capacity of the STZ-icv mucus (Figure 3G,H). The third measure of total redox capacity (ABTS), employed to provide a better understanding of the perplexing discrepancy between ORP and NRP, suggested that there was no pronounced difference (Figure 3I). The lipid peroxidation analysis provided additional evidence for the absence of pronounced (uncompensated) intraluminal redox dyshomeostasis (Figure 3J).

Altered content and functional properties of mucus obtained from the rat model of AD may be associated with decreased barrier-forming capacity.

**Figure 4:**
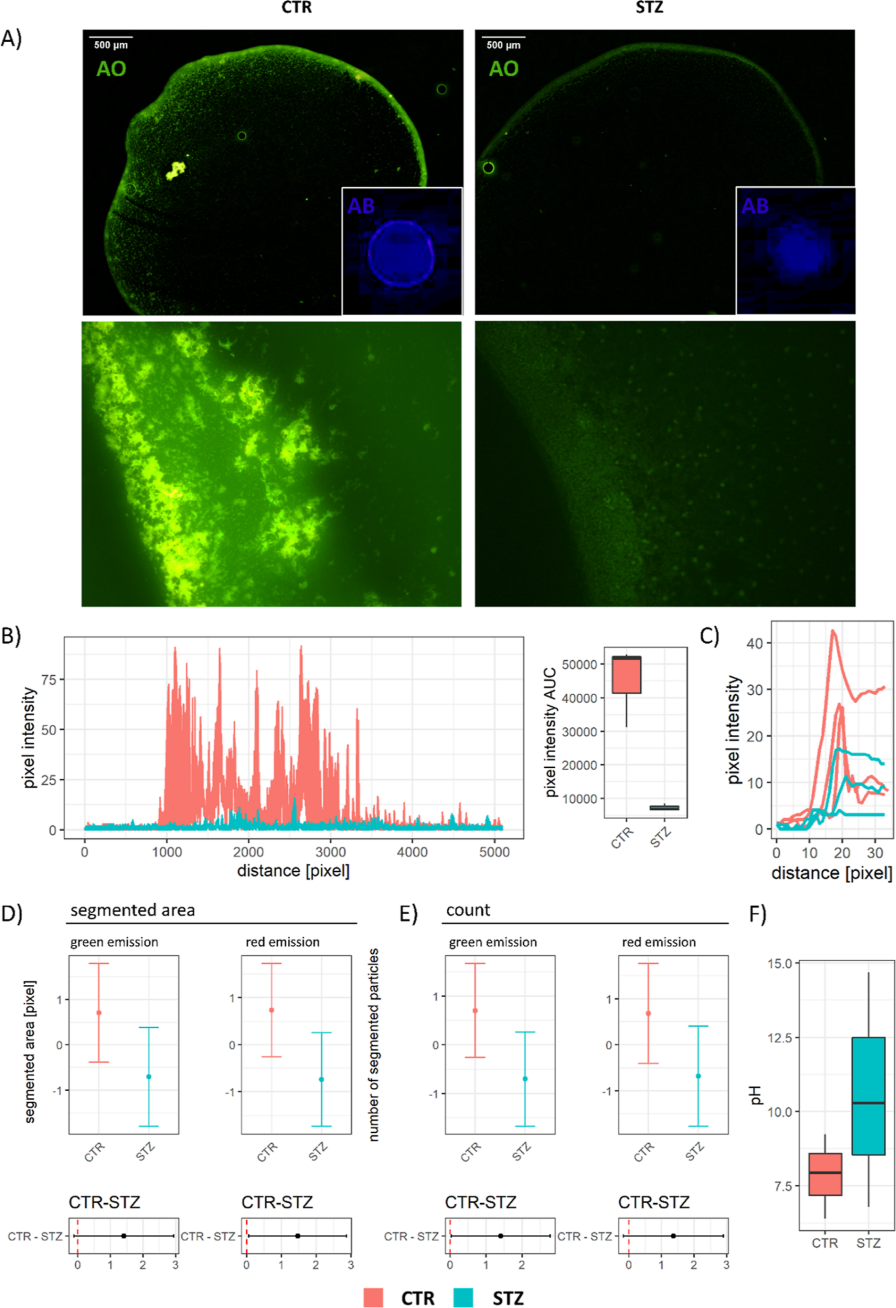
Aggregation and binding of acridinophilic particles in the mucus obtained from the rat model of sporadic AD induced by intracerebroventricular streptozotocin (STZ-icv) and the controls. (A) Representative epifluorescent photomicrographs of AO-stained (large panels) and AB-stained (small inserted panels) mucus showing a prominent aggregation zone in the controls and the absence of thereof in the mucus from the STZ-icv rat model of AD. (B) Line profile plots obtained by quantification of the aggregation zone presented in (A) with the corresponding area under the curve-based intensity calculations. (C) Line profile plots obtained by quantification of the aggregation zone from the AB-stained mucus. (D) Model-derived estimates from the linear model reflecting the total segmented area of the AO signal under two different excitation/emission filter setups (corresponding to eukaryote/prokaryote particle binding) in the mucus of the STZ-icv and the controls (upper) and the contrast illustrating the effect size (lower). Mean estimates are accompanied by 95% confidence intervals. (E) Model-derived estimates from the linear model reflecting the count of AO signal segmented particles under two different excitation/emission filter setups (corresponding to eukaryote/prokaryote particle binding) in the mucus of the STZ-icv and the controls (upper) and the contrast illustrating the effect size (lower). Mean estimates are accompanied by 95% confidence intervals. (F) Boxplots demonstrating the pH of the mucus obtained from the STZ-icv rat model of AD and the controls.

An altered mucus constitution may result in a diminished capacity to form a protective biological barrier. To test the capacity of the CTR and STZ-icv mucus to form barrier-like structures in vitro, the samples were deposited onto microscope slides, and their passive capacity to form aggregates and bind mucus constituents was estimated with morphometric analysis of acridine orange (AO) and AB binding (Figure 4). The control mucus demonstrated a greater capacity to form aggregates and bind acridinophilic substances (Figure 4A), particularly in the peripheral zone, in which a similar pattern was observed following AB staining (Figure 4A–C). The analysis of spectrally decomposed fluorescence intensity (Figure 4D) (reflected by segmented area following thresholding) and signal count (Figure 4E) revealed that CTR mucus has a greater capacity to bind acridinophilic particles of both eukaryote (green fluorescence at pH 3.5) and prokaryote (red fluorescence at pH 3.5) origin. Mucus pH was increased in the STZ-icv, providing one possible explanation for the differences in aggregation and binding capacity and discrepant redox biomarker results (Figure 4F).

The diminished capacity of the STZ-icv mucus to form a functional barrier may be associated with enhanced penetration of small molecules across the mucosal barrier.

**Figure 5:**
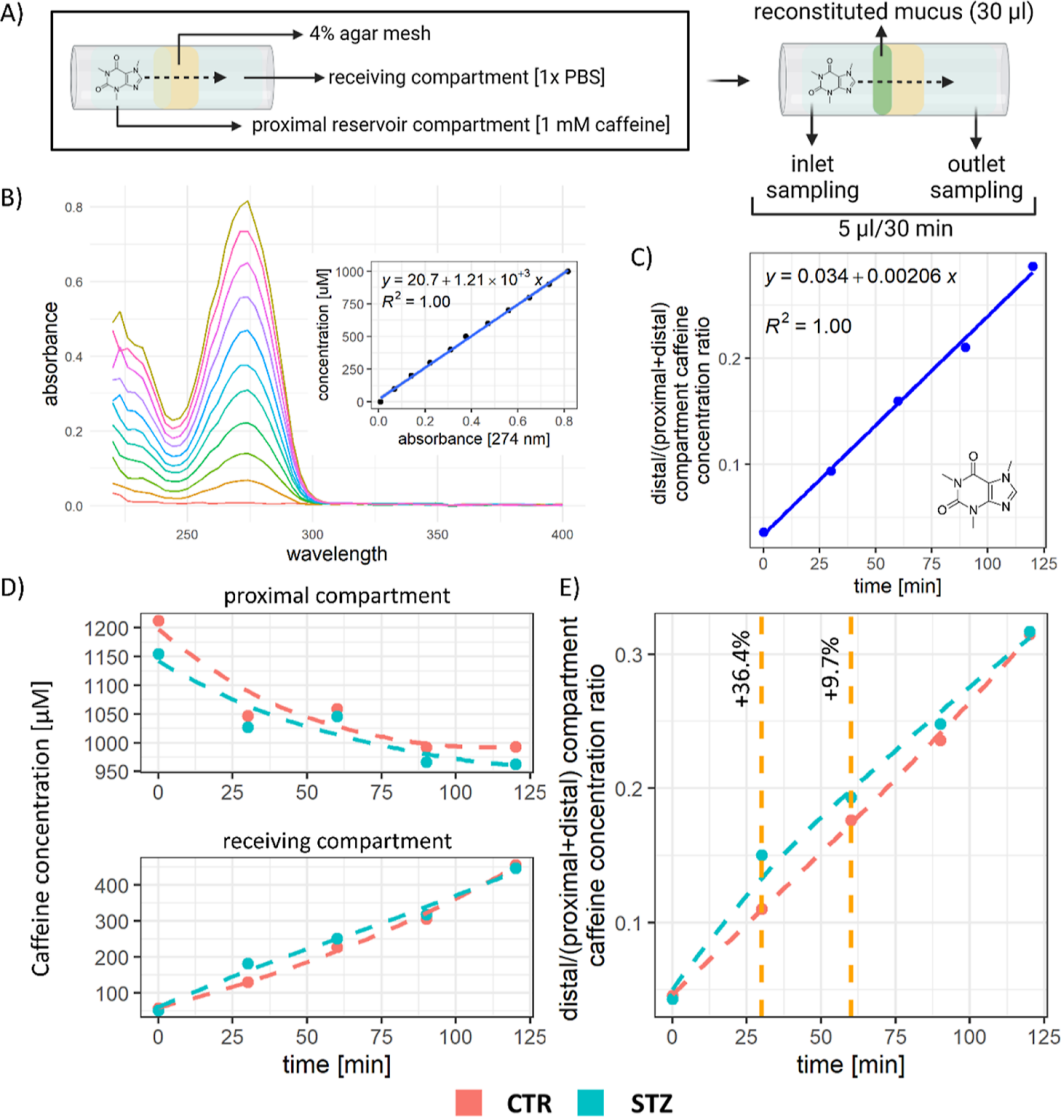
In vitro reconstituted mucus-agar membrane diffusion assay for the assessment of the effect of the mucus barrier on the diffusion of small molecules. (A) Schematic representation of the assay. (B) UV–vis profile of different concentrations of caffeine in phosphate-buffered saline (PBS). (C) Model of caffeine diffusion across the agar barrier. (D) Models reflecting the concentration of caffeine in the proximal and distal compartments of the diffusion chamber. (E) Model of caffeine diffusion across the mucus-fortified agar barrier and the comparison of diffusion profiles in chambers with reconstituted mucus from the STZ-icv rat model of AD and the controls.

Finally, the ability of the CTR and STZ-icv mucus to prevent diffusion of caffeine (used here as a representative small molecule) across the reconstituted barrier was tested using the in vitro agar membrane diffusion assay (Figure 5A) combined with the absorbance-based caffeine concentration model (Figure 5B), which provided a solid framework for the analysis of diffusion interference (Figure 5C). The concentration of caffeine in the receiving compartment increased faster in the container with the reconstituted STZ-icv mucus (Figure 5D,E). The difference in caffeine diffusion was most pronounced in the first two time points (+36.4% [30 min]; +9.7% [60 min]), while both the concentration and diffusion reached a steady state after ∼120 min (Figure 5D,E).

## Discussion

The presented results indicate that GI mucus secretion, constitution, and functional properties are altered in the STZ-icv rat model of AD. Considering that the mucus barrier is integral to gut health, it is reasonable to assume that the reported results may have important implications for (i) understanding the pathophysiological mechanisms responsible for the progressive AD-like phenotype in the STZ-icv model; (ii) elucidating a complex role of the brain–gut axis in the maintenance of GI homeostasis; and (iii) the design of preclinical experiments utilizing the STZ-icv model (e.g., by taking into account a possible difference in the rate of absorption/exposure to some orally administered drugs).

The initial observation that the STZ-icv GI epithelium is characterized by an increased number of mucus-producing goblet cells (Figure 1) led to the hypothesis that goblet cells (i) are produced at a greater rate from the pluripotent stem cells at the base of the crypts (not very likely considering that the villus-crypt ratio was reduced in the STZ-icv^16^); (ii) die off and/or shed off less (e.g., due to altered apoptosis along the villus axis in the STZ-icv epithelium^16^); or (iii) have altered regulation of mucus secretion and/or mucigen secretory vesicle expulsion. The morphometric analysis indicated that an apparent increase in the number of goblet cells may be a reflection of altered mucus secretion as mucigen granules were further away from the epithelial surface and the probability of mucus expulsion was reduced in the STZ-icv (Figure 1D,E).

Goblet cell secretion is regulated by mucus secretagogues that orchestrate exocytosis via modulation of intracellular Ca^2+^.^14,35^ Ach is the most well-studied secretagogue that rapidly stimulates mucus secretion following the release from the neurons of the submucosal plexus.^14,35^ To test whether the STZ-icv goblet cells had altered expulsion of mucigen secretory vesicles, duodenal tissue was incubated with ACh and CCh in concentrations that were previously demonstrated to be sufficient to mimic neurally evoked mucus secretion in the rat intestine.^36^ While the incubation with both ACh and CCh elicited pronounced mucus secretion in the duodenal specimens isolated from the controls, the response was much less pronounced in the STZ-icv goblet cells (Figure 2). The absence of the response in the presence of a reversible competitive antagonist (ATR) in the control group confirmed that mucus secretion was mediated by activation of muscarinic Ach receptors in the CTR; however, in the STZ-icv, co-incubation with a muscarinic antagonist induced a small but consistent potentiation of cholinergic stimulation-mediated mucus secretion via a mechanism that remains to be resolved (Figure 2). The incubation of tissue with CCh was used to indirectly assess the dependence of the observed effects on the activity of Ach esterases (AchE) because of CCh insensitivity to AchE-mediated hydrolysis. In the controls, the effects of ACh and CCh were comparable, suggesting no pronounced effect of endogenous AchE; however, in the STZ-icv, CCh stimulation acted as a more potent stimulus of mucus secretion both alone and in the presence of ATR (in comparison with Ach), suggesting that basal AchE activity may be increased in the STZ-icv gut. The underlying biological mechanism responsible for the observed insensitivity to cholinergic stimulation has yet to be determined. In the brain-first model of Parkinson’s disease, induced by 6-hydroxydopamine in rats, there is a disruption in the fusion of mucin granules with the apical membranes of goblet cells. This is attributed to reduced levels of Ach and increased expression of muscarinic receptor 2. Despite these findings, when exogenous Ach was administered to the STZ-icv goblet cells, it did not stimulate mucus secretion. This suggests that the observed dysfunctional mucus secretion is unlikely to be caused by reduced Ach levels. However, it is important to consider that dysfunctional signaling at the level of muscarinic receptors cannot be ruled out and warrants further investigation.

Considering that goblet cells are responsible for the maintenance of the structure and function of the protective mucus barrier, diminished responsiveness of the STZ-icv goblet cells to cholinergic stimulation may result in qualitative and quantitative alterations in mucus composition with consequences for the GI barrier.^14,35,37^ The analysis of GI mucus composition was in line with reduced responsiveness of goblet cells to cholinergic stimulation in the rat model of AD as the STZ-icv mucus was more diluted (as evident from the reduction in full-spectrum absorbance; Figure 3A), had less alcianophilic (i.e., glycoprotein) and MUC2 content (Figure 3E,F), and had reduced lubrication capacity (Figure 3G). The near-UV region of the STZ-icv mucus showed an interesting pattern of an inverse correlation between the ∼400 nm peak (possibly reflecting the presence of a tetrapyrrole molecule—e.g., bilirubin) and the UV region of the spectrum (Figure 3C), suggesting possible alterations in bile-producing pathways in the STZ-icv rat model of AD. Considering that recent evidence supports the involvement of synthesis and metabolism of bile acids in the pathogenesis of AD (e.g., refs (38)–^39^^40^^41^), future research should focus on the exploration of bile and liver pathophysiology in the STZ-icv rat model of AD.

Based on the results from previous research showing altered redox homeostasis in the STZ-icv GI tract,^15,16^ redox homeostasis of mucus was assessed with the hypothesis that the intraluminal mucus redox state may reflect intestinal changes. Interestingly, the analysis of three different redox biomarkers reflecting total antioxidant capacity (ORP, NRP, and ABTS) provided discrepant results (Figure 3H–J) with no clear evidence of mucus redox dyshomeostasis. A lipid peroxidation marker, TBARS, was also found unchanged, suggesting that: (i) an endogenous factor (e.g., the dilution or pH) may be interfering with reliable estimation of the mucus redox state (most likely); or (ii) mucus redox state does not faithfully reflect the pathophysiological events in the GI wall present in the STZ-icv.^15,16^ For example, NRP indicated that total antioxidant capacity was reduced in the STZ-icv mucus (−30%; *p* = 0.014); however, the difference was less pronounced when the whole spectrum absorbance (i.e., a negative indicator of dilution) was introduced as a covariate (−22%; *p* = 0.089).

Altered mucus content may affect its ability to form a functional barrier and protect the mucosa against intraluminal microorganisms and toxins.^42^ In the STZ-icv mucus, spontaneous formation of protein aggregates was reduced (possibly as a consequence of dilution, reduced content of mucus glycoproteins, and increased pH) and associated with impaired capacity to bind acridinophilic particles (Figure 4). Although in vitro reconstitution of mucus probably only partially reflects its in vivo properties, present results indicate that the mucus layer in the STZ-icv GI tract may be characterized by a diminished capacity to form a barrier to intraluminal microorganisms.

Dynamic alterations of the intraluminal environment affect the function of GI mucus by altering its structural properties. Intraluminal ion concentration and pH regulate the permeability of the GI mucus layer and affect the penetration of nutrients and microorganisms.^42^ Low mucus pH promotes the formation of aggregates and stimulates the assembly of a functional barrier that prevents bacterial transmigration. For example, Sharma et al. have shown that the formation of aggregates was prevented when the pH of the extracted porcine small intestinal mucus was increased from 4 to 7.^42^ Accordingly, reconstituted mucus significantly decreased the transmigration of *Escherichia coli* and *Salmonella enterica* when the pH was decreased from 7 to 4 (or less).^42^ The mucus isolated from the STZ-icv GI tract had a significantly greater pH when compared to that obtained from the control animals (Figure 4F), providing a possible explanation for the observed reduction in the capacity to form glycoprotein-rich aggregates with the capacity to bind acridinophilic particles (e.g., microorganisms) (Figure 4). A significant increase in the STZ-icv mucus pH may also explain some other measurements, as it is reasonable to assume that it could have influenced the assessment of lubrication capacity, redox biomarkers, and even mucus spectral properties.

Finally, qualitative and quantitative alteration of mucus constituents and factors that affect its capacity to form a functional barrier to microorganisms (e.g., pH) may also influence its porosity and molecular permeability. Consequently, the GI mucosa of the STZ-icv rats may be exposed to a greater concentration of intraluminal toxins and some drugs.^43−45^ The results obtained from the in vitro experiment designed for the assessment of the ability of reconstituted mucus to prevent the diffusion of caffeine indicate that the STZ-icv mucus has a reduced capacity to bind and/or prevent the diffusion of small molecules (Figure 5). The observed results should be translated to other small molecules with caution (as only caffeine diffusion was tested because the samples were available only in limited quantities); however, it can be postulated that altered constitution and pH of the STZ-icv mucus may result in generally increased porosity of the GI mucus layer in the rat model of AD. For example, de Moraes et al. recently reported increased absorption of thiamine from the GI tract of STZ-icv rats,^46^ a phenomenon that may be explained by the alteration of the GI mucus layer reported here.

Further research should prioritize the investigation of the precise pathophysiological mechanisms that lead to the disruption of mucus homeostasis in the STZ-icv model. A potential cause of the loss of goblet cell responsiveness and mucus secretion could stem from either local alterations in the GI tract of the STZ-icv model or changes in the functioning of the brain–gut barrier. Previous studies have provided evidence supporting both functional and structural modifications in the gut of the STZ-icv model, as well as changes in the brain–gut axis functionality.^15,47^ Conducting future experiments to gather additional data on the pathophysiological aspects discussed in our study would greatly contribute to a better understanding of the etiopathogenesis of mucus system dysfunction. For instance, as the vagus nerve plays an important role in the efferent control of mucus secretion,^48,49^ incorporating vagotomy experiments could provide crucial insights into the role of brain–gut communication, or the lack thereof, in relation to the functioning of the GI mucus system in the STZ-icv model.

Notably, recent preliminary experiments have also revealed an intriguing finding where Aβ accumulation in the gut of the STZ-icv model precedes its accumulation in the brain. Therefore, it is crucial to consider the possibility that some of the observed changes may also be mediated by GI amyloid.^17^

Additionally, it is important to explore whether restoring the normal function of the GI barrier could potentially mitigate the progression of AD-like pathophysiology. Notably, our recent experiments indicate that the neuroprotective effects of chronic oral d-galactose^50^ may be, at least partially, attributed to alterations in the gut microbiome and the content of short-chain fatty acids (SCFAs).^51^ In the STZ-icv rat model of AD, there was a decrease in the expression of MUC2 (Figure 3), and it is well-established that the microbiota plays a significant role in regulating mucus secretion as well as the expression of MUC2,^52,53^ the primary mucin produced by intestinal epithelial cells. Importantly, SCFA-induced secretion of mucus can be prevented with ATR, suggesting that mechanisms might be mediated by Ach.^54^

## Conclusions

**Figure 6:**
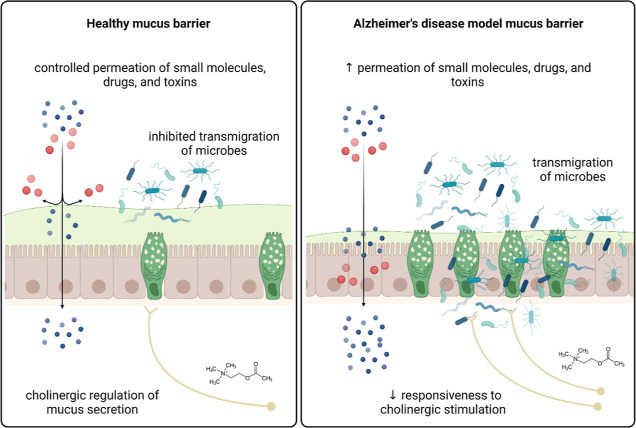
Schematic representation of mucus alterations in the rat model of AD induced by intracerebroventricular streptozotocin (STZ-icv). The GI mucus layer is integral to the maintenance of gut health as it protects the mucosa against direct exposure to intraluminal microorganisms, toxins, and drugs. In the control animals, the structure and function of the mucus barrier are maintained by cholinergic modulation of goblet cell mucus secretion; however, the goblet cells from the STZ-icv rats demonstrate reduced responsiveness to cholinergic stimulation. Consequently, mucus secretion and its structural and functional properties (e.g., binding of microorganisms, lubrication, and permeation of small molecules) are altered in the STZ-icv model of AD, resulting in possibly increased susceptibility to GI and systemic inflammation induced by intraluminal noxious stimuli.

The GI tract of the STZ-icv rat model of AD is characterized by an increased number of mucous-containing secretory vesicles and reduced mucus secretion, possibly caused by decreased responsiveness of goblet cells to cholinergic stimulation. The altered biochemical constitution of the STZ-icv mucus and increased pH are associated with a reduced capacity to lubricate, form glycoprotein aggregates, and bind alcianophilic particles (e.g., microorganisms) (Figure 6). Furthermore, reconstituted STZ-icv mucus shows greater permeability to small molecules. In conclusion, the mucus barrier in the GI tract of the rat model of AD shows structural and functional alterations that may result in greater exposure to intraluminal microorganisms, drugs, and toxins. Consequently, the STZ-icv rat model of AD may be more susceptible to GI and systemic inflammation induced by intraluminal noxious stimuli. Possible pharmacokinetic differences should be anticipated and experimentally evaluated for some orally administered drugs.

## Limitations

There are several important limitations that need to be considered in our study. First, the sample size of animals used in the study is relatively small, which increases the risk of false nondiscovery. Therefore, it is possible that the lack of observed mucus redox changes between the STZ-icv group and the control group could be attributed to small effects that are obscured by the inherent variability in the data. Moreover, the findings obtained from ex vivo and in vitro experiments should be further validated through in vivo experiments. For instance, it would be valuable to quantify the mucus secretion response to cholinergic drugs and measure drug permeability in vivo to confirm our observations. Lastly, it is crucial to conduct more comprehensive experiments to assess the mucus binding capacity, taking into consideration the potential qualitative and quantitative alterations in gut microbiota within the STZ-icv model. By incorporating these factors, we can obtain a more nuanced understanding of the complex interactions involved.

## Materials and Methods

### Animals

Three month-old male Wistar rats bred at the animal facility at the Department of Pharmacology (University of Zagreb School of Medicine, Zagreb, Croatia) were used in the study (experiment 1 [*n* = 8]; experiments 2 and 3 [*n* = 6]). The animals were kept 3 per cage in a controlled environment (room temperature: 21–23 °C; relative humidity: 40–70%); tap water and standardized pellets (Mucedola S.R.L., Italy) were available ad libitum, and standardized bedding was changed twice per week. The light–dark cycle was 7 AM/7 PM. All procedures involving animals were approved by the Croatian Ministry of Agriculture (EP 186 /2018) and the Ethical Committee of the University of Zagreb School of Medicine (380-59-10106-18-111/173).

### Streptozotocin Treatment and Tissue Collection

STZ-icv was used to model AD following a standard procedure originally introduced by Salkovic-Petrisic and Lackovic,^55^ and Hoyer and colleagues.^56−58^ For a detailed overview of the model, please see.^56,57^ Briefly, the animals were randomly stratified into two groups and anesthetized with intraperitoneal ketamine (70 mg/kg) and xylazine (7 mg/kg). The skin was surgically opened, and the skull was trepanated with a high-speed drill bilaterally. The control animals (CTR) were bilaterally intracerebroventricularly administered with vehicle (2 μL/ventricle; 0.05 M citrate buffer; pH 4.5), and the experimental group (STZ) received 1.5 mg/kg STZ by the same procedure first described by Noble et al.^59^ The coordinates were: −1.5 mm posterior; ± 1.5 mm lateral; +4 mm ventral from the pia mater relative to the bregma. The whole procedure was repeated after 48 h so that the cumulative dose of STZ-icv was 3 mg/kg as standardly used.^23,56,57^ After 4 weeks, both groups received 2 μL of sterile saline in each lateral ventricle, as they were used as the controls in the experiment in which the effect of the central administration of [Pro^3^]-GIP was studied.^16^ Successful induction of the model was confirmed by performing standardized tests used for the assessment of aversive and spatial memory following previously described protocols (Supporting Information).^60^ The animals were anesthetized (70 mg/kg ketamine; 7 mg/kg xylazine) and subjected to transcardial perfusion with saline followed by 4% paraformaldehyde (pH 7.4). Duodenal tissue (post-gastric 2 cm) was fixed in 4% paraformaldehyde, dehydrated, and embedded in paraffin blocks.

### Ex Vivo Stimulation of Mucus Secretion

An ex vivo experiment was performed with duodenal tissue samples obtained from 3 control and 3 STZ-icv-treated animals 8 weeks following model induction. The animals were decapitated under deep general anesthesia induced by intraperitoneal administration of ketamine (70 mg/kg) and xylazine (7 mg/kg). A segment of the proximal duodenum was dissected and washed with Krebs solution (115 mM NaCl, 25 mM NaHCO_3_, 2.4 mM K_2_HPO_4_, 1.2 mM CaCl_2_, 1.2 mM MgCl_2_, 0.4 mM KH_2_PO_4_, and 10 mM glucose bubbled with Carbogen (95% O_2_; 5% CO_2_) for 30 min and pre-heated to 35 °C) with a syringe to remove intraluminal contents. The duodenum was placed on top of a wet cellulose paper towel in a Petri dish filled with Krebs solution. 5 (∼4 mm thick) duodenal rings were cut and placed in a 96-well plate pre-filled with (i) Krebs solution (CTR); (ii) Krebs solution + Ach (20 μM); (iii) Krebs solution + Ach (20 μM) + atropine (ATR; 100 μM); and (iv) Krebs solution + carbachol (CCh; 20 μM); (v) Krebs solution + CCh (20 μM) + ATR (100 μM).^36^ After 30 min of incubation, the rings were placed in 4% paraformaldehyde (pH 7.4) and stored at 4 °C. Duodenal rings were cut along the median line and embedded in the water-soluble cryosectioning matrix, Tissue-Tek O.C.T. (Sakura, Japan). Tissue sections (7 μm) were cut using the Leica CM1850 cryosectioning device (Leica Biosystems, Germany).

### Collection of Intestinal Mucus

Intestinal mucus was collected from the same animals used for in vitro cholinergic stimulation of mucus secretion. Briefly, 8 cm long sections of the duodenum distal to the segment used for cholinergic stimulation were removed and washed with Krebs solution with a syringe to remove intraluminal contents. The tissue was carefully longitudinally opened with scissors to expose the luminal surface and placed in a Petri dish. The Krebs solution (1 mL) was pipetted onto the luminal surface, and a clean microscope slide was used to gently scrape the mucus. The Krebs solution was collected from the Petri dish with a pipette and placed back on the luminal surface. The same procedure was repeated five times, and the collection procedure lasted 10 min. The collected mucus was stored at −20 °C. The supernatant collected after 30 min of centrifugation at 10,000*g* was used for the tribometric and biochemical analyses.

### Assessment of the Lubricating Properties of Mucus

Lubrication capacity was assessed with a quantitative tribometric assay described in detail in ref (61). Briefly, a custom-made multifunctional adapter for screening tribometry (mastPASTA)^61^ was connected to the PASTA platform^62,63^ to enable the acquisition of time series data of the vertical pulling force applied to the polyvinyl chloride (PVC) tubing pre-treated with collected mucus. The mucus (10 μL) was administered at the concave surface of the outer tubing and spread over the proximal ∼188.5 mm^2^ with repeated (*n* = 10) rotating insertions. The outer tubing was connected to mastPASTA with a pin, and 10 vertical pulls corresponding to the contact surface of 125.66 mm^2^ were recorded. The PASTA-derived values were multiplied by the acceleration of gravity (9.80665) to obtain the force in mN, and the peak force for each pull was extracted.

### AB Staining

AB 8GX powder (Sigma-Aldrich, USA) was dissolved in 200 mL of 3% v/v acetic acid in distilled water to obtain a 1% w/v solution. The solution was filtered, and the pH was adjusted to 2.5. The slides containing formalin-fixed paraffin-embedded tissue from the in vivo experiment were deparaffinized, while cryosections from the ex vivo experiment were equilibrated in 1× PBS for 15 min. The tissue was hydrated and incubated in an AB solution for 15 min. The sections were washed in running tap water, rinsed in distilled water, and cover-slipped with a Fluoroshield mounting medium containing DAPI (Abcam, UK).

### UV–Vis Spectrophotometry

The UV–vis spectra (220–750 nm) were measured with the NanoDrop ND-1000 (Thermo Fisher Scientific, USA). Mucus spectra were obtained by scanning 1 μL of mucus supernatant. 230 nm was used as an indicator of salt content, 260 nm absorbance was extracted as an indicator of DNA/RNA content due to the aromatic base moieties within their structures, and 280 was used as an indicator of protein content due to aromatic amino acid side chain absorbance in this range.

### Protein Quantification

Considering that phenol groups of some organic compounds can also absorb light at 280 nm, protein content was additionally measured using the Bradford assay (Sigma-Aldrich, USA). Bovine serum albumin dissolved in 1× PBS was used for the standard curve. The absorbance at 595 nm was quantified using the Infinite F200 PRO multimodal microplate reader (Tecan, Männedorf, Switzerland).

### AB Dot Blot

AB dot blot was performed for the quantification of mucin content in the supernatant of isolated mucus samples. Briefly, 2 μL of each sample was deposited onto the Superfrost Plus Gold Adhesion Microscope slides, heated to 40 °C, and incubated in a 1% AB solution for 15 min. The slide was washed in dH_2_O to remove the residual dye and left to air dry. The slide was digitalized and quantified in Fiji using integrated density obtained with the gel analyzer algorithm.

### Dot Blot Quantification of MUC2

Mucus proteins were precipitated by incubating 50 μL of sonicated mucus with 200 μL of ice-cold acetone for 1 h at −20 °C. The supernatant was removed, the remaining acetone was evaporated at room temperature, and the pellet was briefly sonicated in 20 μL of sample buffer containing 40% glycerol, 8% SDS, and 200 mM Tris HCl. Protein extracts (1 μL) were spotted onto the nitrocellulose membrane, dried, and incubated in blocking buffer (5% nonfat-dried milk, 0.5% Tween-20, 10 mM Tris, 150 mM NaCl; pH 7.5) for 1 h at room temperature to prevent non-specific antibody binding. Blocked membranes were incubated with the rabbit anti-MUC2 primary antibody (Boster Biological Technology, USA) diluted in the blocking buffer 500-fold for 24 h at 4 °C. Membranes were washed three times in low-salt washing buffer (LSWB; 10 mM Tris, 150 mM NaCl; pH 7.5) and incubated with goat anti-rabbit poly-HRP secondary antibody (Thermo Fisher Scientific, USA) diluted in the blocking buffer (1:500) at room temperature for 2 h. Membranes were washed in LSWB and incubated in a chemiluminescence reagent (SuperSignal West Femto; Thermo Fisher Scientific, USA). The signal was recorded with a MicroChemi high-performance chemiluminescence imager (DNR Bio-Imaging Systems, Israel) and analyzed in Fiji (NIH, USA) using the gel analyzer plugin for calculating dot blot integrated signal density.

### Biochemical Analysis of Redox Biomarkers

The ORP, NRP, and ABTS radical cation assays were used to measure redox mucus potential, and the TBARS assay was used to quantify the end products of lipid peroxidation. The ORP was measured using the 6230N Microprocessor meter (Jenco Instruments, San Diego, USA) connected to an ORP-146S redox microsensor (Shelf Scientific, Lazar Research Laboratories, USA) with a platinum sensing element, Ag/AgCl reference, and KCl filling solution.^64^ A higher ORP value indicates a greater oxidative potential, reflecting increased oxidative stress.^65^ NRP utilizes sample-mediated KMnO_4_ reduction in the pH-neutral environment on the nitrocellulose membrane to capture solid MnO_2_ precipitates that can then be quantified for the assessment of total reductive capacity.^31^ 1 μL of each mucus sample was pipetted onto the nitrocellulose membrane (Amersham Protran 0.45; GE Healthcare Life Sciences, Chicago, IL, USA), left to dry out, immersed in a NRP reagent (0.2 g KMnO_4_ in 20 mL ddH_2_O) for 30 s, and destained under running dH_2_O. The dry membrane was digitalized and analyzed in Fiji using the gel analyzer plugin, as described in detail in ref (31). The ABTS cation radical assay was done by first reacting 7 mM ABTS with 2.45 mM K_2_S_2_O_8_ overnight to generate the ABTS radical cation.^66^ The ABTS radical cation solution was diluted at 1:40 to obtain the ABTS working solution. 1 μL of each sample was incubated with 100 μL of the ABTS working solution, and the absorbance at 405 nm was measured after 300 s using an Infinite F200 PRO multimodal microplate reader (Tecan, Switzerland). Serial dilutions of the reducing agent 1,4-dithiothreitol were used for the generation of the standard model.^67^ TBARS assay was used for the assessment of lipid peroxidation. Briefly, 20 μL of each sample was incubated with 70 μL of ddH_2_O and 140 μL of the TBARS reagent (0.4% w/v thiobarbituric acid dissolved in 15% w/v trichloroacetic acid) at 95 °C for 90 min in perforated microcentrifuge tubes. The colored adduct was extracted in 100 μL of *n*-butanol, and the absorbance was measured at 540 nm in a 384-well plate using the Infinite F200 PRO multimodal plate reader. MDA tetrabutylammonium (Sigma-Aldrich, USA) was used for the generation of the standard model.

### AO Staining

AO solution (pH 3.5) was prepared by dissolving 20 mg of AO powder in 190 mL Na-acetate buffer (100 mL of 1 M *N*-acetate trihydrate + 90 mL 1 M HCl).^68^ The slides (Superfrost Plus Gold Adhesion) with fixed mucus samples were immersed in the AO solution for 2 min, rinsed in tap water and dH_2_O, and cover-slipped with the Fluoroshield mounting medium. AO mucus staining was analyzed using the U-MNIB2 and U-MWIG2 filter sets on the Olympus BX51 epifluorescent microscope (Olympus, Japan).

### Mucus pH Measurement

As mucus volume was too low to be estimated using a standard pH probe, a simple thymolsulfonephthalein assay was established. Briefly, 100 mg of thymolsulfonephthalein was dissolved in NaOH ethanol solution (2.15 mL of 0.1 M NaOH in 20 mL of 95% ethanol), and the reaction buffer was diluted to 100 mL total volume with ddH_2_O. The pH-adjusted (with HCl and NaOH) PBS was used for the generation of the calibration curve. 2 μL of the standard solution or the sample was mixed with 8 μL of the thymolsulfonephthalein working reagent. The colorimetric shift was recorded with a camera (Samsung S20FE, Samsung, Suwon-si, South Korea) and NanoDrop ND-1000 (Thermo Fisher Scientific, USA).

### Caffeine Diffusion Model

The caffeine diffusion model was used to test the effect of different mucus constitutions on the diffusion rate of small molecules. Caffeine was chosen due to its small size (estimated average mass of 194.191 Da) and a distinct 275 nm peak. Agar agar (4% w/v in ddH_2_O) was used as a supporting structure to enable the reconstitution of mucus on a solid surface due to its permeability to water and caffeine, low chemical reactivity (commonly used as a drug stabilizer), and easy manipulation (e.g., the thickness of the membrane and its porosity can be easily modified by altering the concentration and volume). Briefly, the proximal reservoir and the receiving compartment were created by cooling 60 μL of the 4% w/v agar solution inside a custom-made PVC container (length: 20 mm; total volume: 392 mm^3^) to create two identical compartments with ∼150 mm^3^ capacity divided by a permeable agar gel membrane. The containers were cooled at −20 °C before use. Caffeine diffusion across the agar membrane was first estimated in containers of different sizes using the variable thickness and porosity (concentration) of the agar membrane. The concentration of caffeine was estimated from the quantitative model based on the 274 nm absorbance of serial dilutions in 1× PBS. Once optimal assay conditions have been established, pooled CTR and STZ mucus solutions (30 μL) were deposited on top of the agar gel in proximal reservoir compartments, and the mucus was left to form the membrane for 20 min at room temperature. The caffeine solution (100 μL; 1 mM in 1× PBS) was delivered into the proximal compartment, and the same volume of 1× PBS was added to the receiving compartment. The control conditions included: (i) 1× PBS in both compartments (to control for the effects of elution and diffusion of components from agar); (ii) 1× PBS in both compartments with CTR mucus formed on top of the agar membrane in the proximal compartment (to control for the effects of diffusion of mucus components); (iii) 1× PBS in both compartments with STZ mucus formed on top of the agar membrane in the proximal compartment (to control for the effects of diffusion of mucus components that are qualitatively and/or quantitatively different in the STZ in comparison with the CTR mucus); (iv) a simple caffeine diffusion model (proximal compartment—1 mM caffeine; distal compartment—1× PBS; agar membrane with no mucus). Sampling time points were: 0, 30, 60, 90, and 120 min, and 5 μL of the solution was sampled from each compartment for all the conditions tested at every time point. Spectra were recorded using the NanoDrop ND-1000 (Thermo Fisher Scientific, USA).

### Image Analysis

Morphometric analysis and calculation of estimated segmented AB surface areas were done in Fiji (NIH, Bethesda, USA) using microscopic images obtained by the Olympus BX51 and CellSens Dimensions image acquisition software (Olympus, Japan). Morphometric analysis was done by (i) calculating the number of goblet cells/villus; (ii) measuring the mucosal surface not covered by goblet cells (pixel-length of segmented lines adjacent to mucosal surface connecting the intersections of lines perpendicular to the mucosal surface with the origin in the center of the goblet cell AB-stained vesicles); (iii) calculating the proportion of goblet cells undergoing expulsion (defined as an AB-stained vesicle either touching the mucosal surface or undergoing exocytosis); (iv) calculating the pixel distance of AB + vesicles from the mucosal surface. Mucus content was estimated from the AB surface area by first segmenting the image to obtain AB masks (the color split was performed in inverted images, and an inverted red channel was used for subsequent thresholding using the Renyi entropy algorithm^69^) and then analyzing the masks with the Fiji particle analysis algorithm. Regions of interest (crypt, villus, and luminal area) were defined manually as 240,000 pixel^2^ (800 × 300 pixels) representative anatomical regions. A total of 6609 masks were analyzed, and the sum of the area was calculated as a proxy for mucus content for each sample. The total area of the segmented mucus fluorescent signal and particle count (AO) were obtained by color splitting followed by the triangle thresholding method directed to the particle analysis algorithm in Fiji.

### Data Analysis

Data were analyzed in R (4.1.3) following the guidelines for reporting animal research.^70^ Data from the morphometric analysis of the AB signal from the in vivo study was analyzed as follows: (1) the number of goblet cells/villus was defined as the dependent variable, while the group was defined as a fixed effect in the linear mixed model. Repeated measurements within each animal were accounted for by fitting a nested random effects term. The same approach was used for the estimation of the goblet cell-free area and the distance between the goblet cell mucus and the epithelial surface (both defined as dependent variables in respective mixed effects models). The probability of mucus expulsion was estimated using mixed effects logistic regression with the same nested random effect to account for the hierarchical data structure. The ex vivo mucus expulsion experiment was analyzed by fitting linear mixed effects models to account for a more complicated design with five treatments tested in intestinal rings obtained from each animal previously treated either with vehicle (CTR) or STZ. The sum of segmented areas in pre-defined regions of interest was defined as the dependent variable. The group, treatment, and group × treatment interaction were defined as fixed effects, and the animal from which the tissue was obtained was the random effects variable. The AO fluorescent signal (total area, count) was analyzed by linear mixed models with animals defined as the random effects variable (to account for repeated measurements introduced to increase assay precision). Protein concentration, AB dot blot density, mastPASTA peak force, ORP, NRP, ABTS, and TBARS were analyzed by simple linear regression with group allocation defined as the independent variable. Model assumptions were checked using a visual inspection of residual and fitted value plots. Models were reported as (i) point estimates of least squares means with corresponding 95% confidence intervals, and (ii) contrasts defined as differences of estimated marginal means with accompanying 95% confidence intervals.

## Supporting Information Available

Outcomes of passive avoidance and Morris Water Maze tests, which provide additional evidence supporting the successful establishment of the STZ-icv model (PDF)

## Notes

Raw data can be obtained from the corresponding author. The manuscript has been preprinted on bioRxiv (10.1101/2022.10.03.510623).

## Notes

Ethics Approval: All animal procedures were conducted per institutional (University of Zagreb School of Medicine), national (The Animal Protection Act, NN135/2006; NN 47/2011), and international (Directive 2010/63/EU) guidelines governing the use of experimental animals. The experiments were approved by the Ethical Committee of the University of Zagreb School of Medicine (380-59-10106-18-111/173) and the Croatian Ministry of Agriculture (EP 186 /2018). Animal experiments comply with the ARRIVE guidelines.
