# Supplementary material for: Altered Secretion, Constitution, and Functional Properties of the Gastrointestinal Mucus in a Rat Model of Sporadic Alzheimer’s Disease
Source: ACS Chem Neurosci. 2023 Jul 21;14(15):2667–82. doi: 10.1021/acschemneuro.3c00223 (PMC10401635; doi:10.1021/acschemneuro.3c00223)
Supplement: Supplementary file 1 — cn3c00223_si_001.pdf [file cn3c00223_si_001.pdf]

Supporting Information:

Altered secretion, constitution, and functional properties of the gastrointestinal mucus in a rat model of sporadic Alzheimer's disease

Jan Homolak[1,2], Joke De Busscher[3], Miguel Zambrano Lucio[4], Mihovil Joja[1,5,6],Davor Virag[1,2], Ana Babic Perhoc[1,2], Ana Knezovic[1,2], Jelena Osmanovic Barilar[1,2], Melita Salkovic-Petrisic[1,2]

[1] Department of Pharmacology, University of Zagreb School of Medicine, 10 000 Zagreb, Croatia

[2] Croatian Institute for Brain Research, University of Zagreb School of Medicine, 10 000 Zagreb, Croatia

[3] Catholic University of Leuven, 3000 Leuven, Belgium

[4] Autonomous University of Nuevo Leon, School of Medicine, Monterrey, 66455 Nuevo Leon, Mexico

[5] Department of Infection and Immunity, Luxembourg Institute of Health, L-4354 Esch-sur-Alzette, Luxembourg

[6] Faculty of Science, Technology and Medicine, University of Luxembourg, L-4365 Esch-sur-Alzette, Luxembourg

Corresponding author:

Jan Homolak, MD

Department of Pharmacology,
University of Zagreb School of Medicine,
Šalata 11, 10 000 Zagreb,
Croatia

+385 91 9411 468
Institutional email: jan.homolak@mef.hr

The passive avoidance and Morris Water Maze tests were conducted following established protocols, as previously described (1).

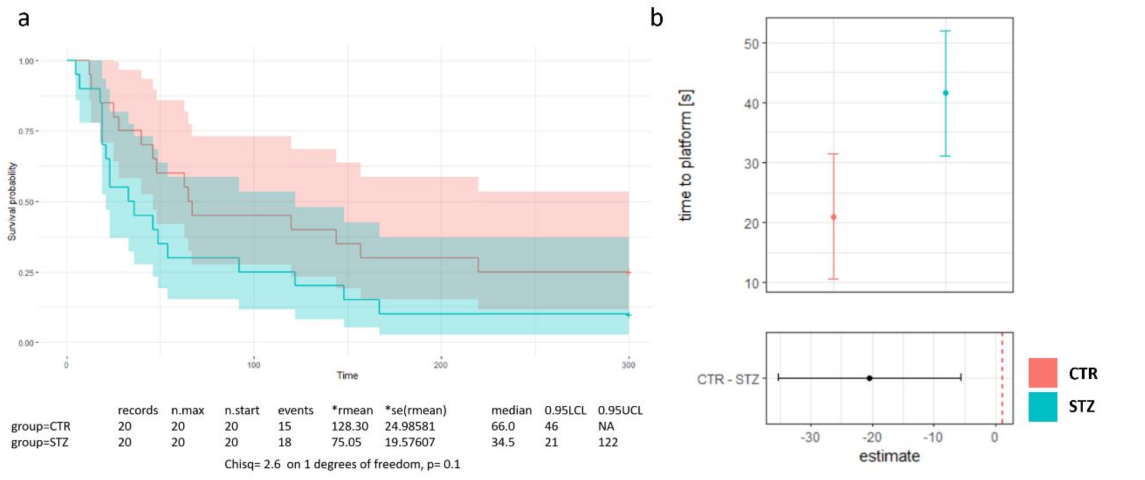

Fig S1 Confirmation of the successful induction of the STZ-icv model, as assessed by cognitive tests measuring aversive memory using the passive avoidance test (A) and spatial memory using the Morris Water Maze test (B). To account for potential ceiling effects, a survival model was employed, as control animals typically reached the maximum time limit of 300 seconds.

1. Knezovic, A., M. Piknjac, J. Osmanovic Barilar, A. Babic Perhoc, D. Virag, J. Homolak, and M. Salkovic-Petrisic. 2023. Association of Cognitive Deficit with Glutamate and Insulin Signaling in a Rat Model of Parkinson’s Disease. *Biomedicines*. 11.
